# Increased extracellular volume after aortic valve replacement: A footprint of reverse ventricular remodeling that does not affect conduction velocity

**DOI:** 10.1016/j.jocmr.2025.101936

**Published:** 2025-08-06

**Authors:** Vladimír Sobota, Christoph M. Augustin, Gernot Plank, Edward J. Vigmond, Sarah Nordmeyer, Jason D. Bayer

**Affiliations:** a IHU Liryc, Electrophysiology and Heart Modeling Institute, Fondation Bordeaux Université, Bordeaux, France; b University of Bordeaux, Institut de Mathématiques de Bordeaux, UMR 5251, Talence, France; c Medical University of Graz, Graz, Austria; d BioTechMed-Graz, Graz, Austria; e Department of Diagnostic and Interventional Radiology, Tübingen University Hospital, University of Tübingen, Tübingen, Germany; f Institute of Computer-Assisted Cardiovascular Medicine, German Heart Center Charité, Berlin, Germany

**Keywords:** Aortic stenosis, Cardiovascular magnetic resonance, Computer modeling and simulations, Conduction velocity, Diffuse fibrosis, Extracellular volume

## Abstract

**Background:**

Extracellular volume ) determined by cardiovascular magnetic resonance (CMR) is considered a marker of diffuse myocardial fibrosis and a predictor of mortality. Using personalized computational models, we investigated the relationship between ECV, conduction velocity (CV), and cell radius in aortic stenosis (AS) patients.

**Methods:**

CMR was performed on 12 AS patients (6 males, 6 females) before and three months after surgical aortic valve replacement (AVR). All patients had a QRS duration ≤110 ms, and no scar on late gadolinium enhanced (LGE) CMR. Computational biventricular models were developed from each CMR dataset. Using patient-specific ECV and the relative change in cell radius between the time points as inputs, tissue conductivity was adjusted in each model to match the patient’s QRS duration. A physiological pattern of ventricular depolarization was mimicked by simultaneously pacing each model from five activation sites. CV was measured during a simulation of apical pacing, using two points positioned at the right ventricular septum of the model.

**Results:**

Left ventricular mass decreased after AVR (62 [58–79] vs 51 [41–60] g/m^2^, p = 0.0005) while ECV increased (24.2 [20.6–24.8] vs 28.0 [25.1–29.5] %, p = 0.0008). No changes in the patient’s QRS duration (89.0 [80.5–99.0] vs 88 [78.5–99.5] ms, p = 0.2148) were observed. No changes in the CV obtained from the models (64.3 [61.9–72.8] vs 66.0 [60.0–74.5] cm/s, p = 0.5186) were found between the time points, suggesting there was no substantial increase in diffuse fibrosis. ECV was negatively correlated with cell radius (r = −0.5267, p = 0.0082), but not correlated with CV obtained from the models (r = −0.2036, p = 0.3399).

**Conclusion:**

Increased ECV three months after AVR in patients with no LGE scar and with normal ventricular conduction appears to be a footprint of reverse ventricular remodeling that does not necessarily translate into changes in myocardial CV.

## Graphical abstract

**Figure:**
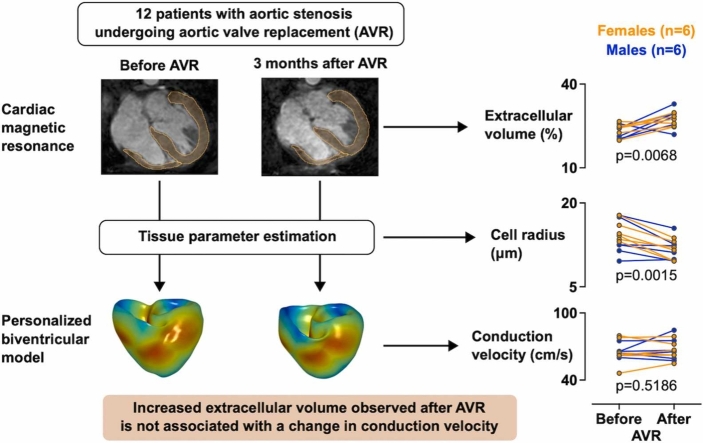

## 1. Introduction

Extracellular volume (ECV) determined by cardiovascular magnetic resonance (CMR) helps to quantify the amount of extracellular space within myocardium [1]. Being linearly proportional to fibrosis from histology [2], ECV is capable of detecting atypical and diffuse myocardial fibrosis that is not visible on late gadolinium enhancement (LGE) CMR images [3]. Increased ECV was reported in aortic stenosis (AS) patients one year after aortic valve replacement (AVR) [4] and was suggested as a predictor of mortality in AS patients [5], [6]. AVR is followed by ventricular remodeling characterized by a reduction in left ventricular (LV) mass that occurs jointly due to the regression of cellular hypertrophy and a decrease in extracellular matrix volume [4], [7]. However, since ECV only captures the volume of non-myocytes without detecting collagen in the extracellular space [8], it cannot determine whether an ECV increase has been caused by a reduction in cellular hypertrophy, an increase in interstitial fluid, or an increased amount of diffuse fibrosis. This makes the identification of the underlying cause of ECV changes challenging without additional structural information.

Histological descriptions showed that post-AVR remodeling is associated with a shortening of muscle fiber diameter and a decrease in fibrous tissue content [9]. However, obtaining myocardial tissue biopsies from post-AVR patients can be ethically complicated, especially when patients have favorable outcomes and are not recommended for an invasive procedure. Characterization of the tissue remodeling in these patients is therefore limited to CMR findings, and the relevance of increased ECV after AVR remains open to interpretation.

An alternative method is necessary to further characterize the remodeling process that follows AVR in patients with favorable outcomes and the role of ECV in it. Expansion of the extracellular matrix volume, presence of collagen in the extracellular space, and reduction of the myocyte size due to a regression of cellular hypertrophy can each, by themselves, alter cardiac electrophysiology. These changes can lead to prolonged ventricular depolarization due to slower ventricular conduction [10], and eventually cause increased vulnerability to ventricular arrhythmias. However, studying changes in each of these tissue parameters simultaneously and in three dimensions is challenging.

Personalized computational models, often referred to as “digital twins,” are a proven tool for studying cardiac electrophysiology at multiple scales and in three dimensions [11]. In this study, we used CMR and electrocardiogram (ECG) data to create ECG-calibrated digital twins of the ventricles of AS patients before and after surgical AVR. By applying a newly developed computational approach that estimates changes in tissue properties while using ECV as an input [10], we studied how post-AVR ventricular remodeling affects tissue properties, particularly the cell radius (R). The models also estimated ventricular conduction velocity (CV) at different time points. By evaluating the association between ECV, R, CV, clinical, and CMR-derived parameters, we provide a further characterization of the reverse ventricular remodeling that follows AVR. We demonstrate that increased ECV after AVR can be considered merely a footprint of the regression of cellular hypertrophy.

## 2. Methods

### 2.1. Patient population

The patients included in this study (n = 12) were diagnosed with severe AS according to current guidelines [12], and were recruited at the German Heart Center Berlin, Germany. Additional details about the patient cohort can be found in a previous study [13]. The following exclusion criteria were applied: the presence of moderate to severe aortic regurgitation, mitral, pulmonary, or tricuspid valve disease, the presence of coronary artery disease, and general contraindication to CMR. To include only patients in whom a physiological pattern of ventricular depolarization could be assumed, we excluded patients with bundle branch block [14], a QRS duration >110 ms, and a scar visible on LGE CMR.

The patients underwent echocardiographic, electrocardiographic, and CMR examinations before and three months after surgical AVR. The study protocol was in agreement with the Declaration of Helsinki and was approved by the Medical Ethics Review Committee. All patients gave written consent prior to being included in the study.

### 2.2. CMR acquisition

The CMR examinations were performed using a whole body 1.5T CMR system (Achieva R 3.2.2.0, Philips Healthcare, Best, The Netherlands). Balanced steady-state free precession (bSSFP) cine 2-dimensional short-axis sequences, 2- and 4-chamber views, were obtained using a previously applied CMR protocol for LV volumetric and anatomical measurements [15]. Analysis was performed using View Forum (R6.3V1L7 SP1, Philips Healthcare). LV epicardial and endocardial borders were manually drawn in diastole and systole to obtain LV volume, end-diastolic volume, and end-systolic volume, and to calculate ejection fraction. In addition, bSSFP 3-dimensional images were obtained during end-diastole (3 signal averages, navigator gated, ECG triggered, reconstructed voxel size of 0.8 × 0.8 × 2.0 mm) and were used for generating digital twins of the human ventricles.

LGE images were acquired 10–15 min after bolus injection of 0.2 mmol/kg gadobenate meglumine (Dotarem; Guerbet) with an inversion-recovery 3-dimensional spoiled gradient echo sequence. Typical parameters were a voxel size of 1.7 × 1.7 × 5.0 mm^3^, a repetition time/echo time = 3.3/1.6 ms, and a flip angle of 15°. Short-axis LGE views of the entire LV myocardium and 2-, 3-, and 4-chamber LGE views were obtained. [16].

ECV was calculated from T1 values from native and 15 min post-contrast T1-mapping using a modified Look-Locker inversion recovery (MOLLI) 5s(3s)3s-scheme and a hematocrit value that was measured on the day of the CMR scan. Typical imaging parameters were as follows: an acquired voxel size 2.0 × 2.0 × 10 mm^3^, a reconstructed voxel size 0.5 × 0.5 × 10 mm^3^, a bSSFP readout, flip angle 35°, a parallel imaging (SENSE) factor 2, and effective inversion times between 150 and 3382 ms [16], [17]. Two T1 slices in short-axis view were acquired in each patient at the base and middle of the ventricles. A basal, mid-ventricular, and average ECV was calculated from these slices [16].

### 2.3. Generation of digital twins of human ventricles

**Fig. 1:**
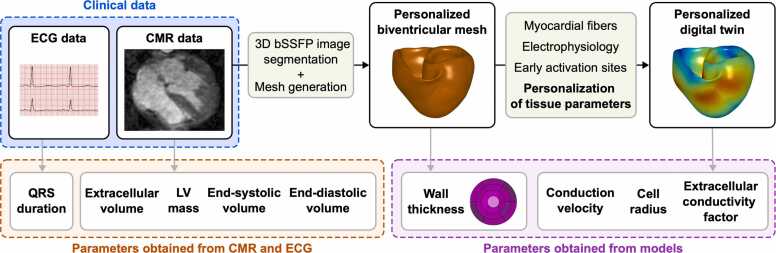
Generation of personalized digital twins of human ventricles from CMR and ECG data. Personalized biventricular meshes were created from bSSFP CMR images. Myocardial fibers, cellular ventricular electrophysiology, and early activation sites were incorporated into the meshes. The bottom part of the figure shows the parameters that were obtained from CMR and ECG data and the parameters that were derived from the computational models. QRS duration was obtained from ECG; extracellular volume, LV mass, end-systolic and end-diastolic volumes were obtained from CMR data. Wall thickness was measured in the biventricular meshes for each AHA segment. Cell radius and extracellular conductivity factor are model parameters that were personalized using the method described in Fig. 3 that uses the patient’s QRS duration as a constraint. Conduction velocity was measured at the RV septum of each model during RV apical pacing (see Supplementary Methods and Fig. S2 for additional details). *CMR* cardiovascular magnetic resonance, *ECG* electrocardiogram, *bSSFP* balanced steady-state free precession, *LV* left ventricular, *RV* right ventricular, *AHA* American Heart Association

Personalized computational models of the human ventricles were created following the general framework for the generation of digital twins [18]. This modeling approach was adopted from previous studies and has been extensively validated using experimental animal and clinical human data [19], [20]. The workflow is schematically illustrated in Fig. 1. Briefly, bSSFP imaging data were segmented in the MUSIC software [21] and Seg3D (http://www.seg3d.org). NumeriCor Studio (https://www.numericor.at) was used to generate tetrahedral volumetric meshes with an average edge resolution of 475 µm. Myocardial fibers were assigned using an established rule-based approach [22]. A physiological pattern of ventricular depolarization [23] was simulated in each model by stimulation from five early activation sites (EAS) [18]. In addition, a stimulation site at the endocardial side of the right ventricular (RV) apex was defined to to simulate RV apical pacing. Human cellular electrophysiology was simulated by the ten Tusscher ventricular myocyte model [24]. Further details regarding the generation of personalized digital twins, including the properties of individual ventricular meshes, are provided in the Supplementary Materials.

### 2.4. Wall thickness measurements

In addition to LV mass assessed by CMR, the reverse ventricular remodeling that follows AVR was quantified by measuring LV wall thickness in the personalized computational meshes derived from CMR data at end-diastole (Fig. 1). Using the universal ventricular coordinate system [25], wall thickness was determined for each American Heart Association (AHA) segment of the LV [26] to assess regional differences in wall thickness and to present them in the form of a bull’s eye plot. Together with ECVs from before and after AVR, the average wall thicknesses from both time points were used to calculate the relative change in cell radius (R). Details on this calculation are provided in the Supplementary Methods.

### 2.5. Personalization of tissue parameters

**Fig. 2:**
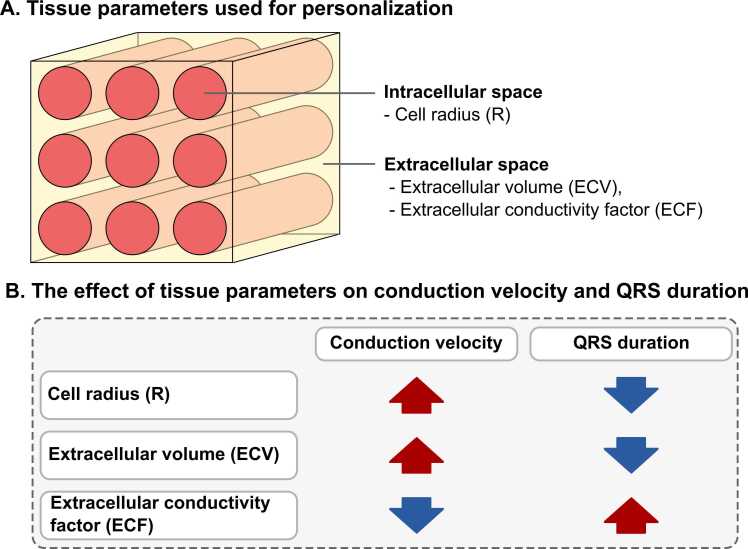
The tissue parameters used for personalization of the models and their effect on CV and QRS duration. (A) R is the parameter that determines the degree of cellular hypertrophy. Extracellular space is characterized by ECV and ECF. ECV describes the volume of extracellular space in the absence of fibrosis, while ECF reflects the amount of fibrotic content in the extracellular space. ECF scales the conductivity of extracellular space and has values between 0% (no fibrosis, i.e., the extracellular conductivity is not altered) and 100% (the extracellular space is non-conductive). (B) R and ECV increase CV and lead to a shortening of the QRS duration. In contrast, ECF decreases CV and prolongs QRS duration. Sensitivity analysis that underlies the effects described in panel B can be found in Supplementary Fig. S4. *CV* conduction velocity, *R* cell radius, *ECV* extracellular volume, *ECF* extracellular conductivity factor

Cellular hypertrophy and tissue fibrosis are factors that substantially affect ventricular conduction [10] and lead to changes in QRS duration. Considering an idealized ventricular tissue with cells represented by cylinders (Fig. 2A), the degree of cellular hypertrophy can be quantified by R. However, myocardial fibrosis is characterized by ECV only partially [8], because ECV captures the volume of non-myocytes, not the actual amount of collagen in the extracellular space. We therefore considered ECV and the amount of fibrosis as two independent variables, defining the extracellular conductivity factor (ECF) as a parameter that indirectly quantifies the amount of extracellular fibrotic content. ECF scales the conductivity of the extracellular space (exact values are provided in Supplemental Table S1), and has values between 0% (no fibrosis, i.e., the extracellular conductivity is not altered) and 100% (the extracellular space is non-conductive). The conductivity of the ventricular tissue in our personalized digital models is therefore determined by R, ECV, and ECF. We do not consider any changes to the active membrane properties of the myocytes between the time points before and after AVR.

The effects of R, ECV, and ECF on ventricular conduction are summarized in Fig. 2B. Cellular hypertrophy, parametrized in our models as an increase in R, accelerates ventricular conduction and leads to a shorter QRS duration. This effect has been observed experimentally [27] and thoroughly studied in computational models [28]. Similarly, an increase in ECV in the absence of fibrosis leads to increased CV and QRS shortening (see Supplementary Methods and Supplementary Discussion for further details). In contrast, increased ECF leads to slower ventricular conduction and prolongs QRS duration. This is due to the presence of collagen in the extracellular space that disrupts the electrical connections between myocytes [29].

**Fig. 3:**
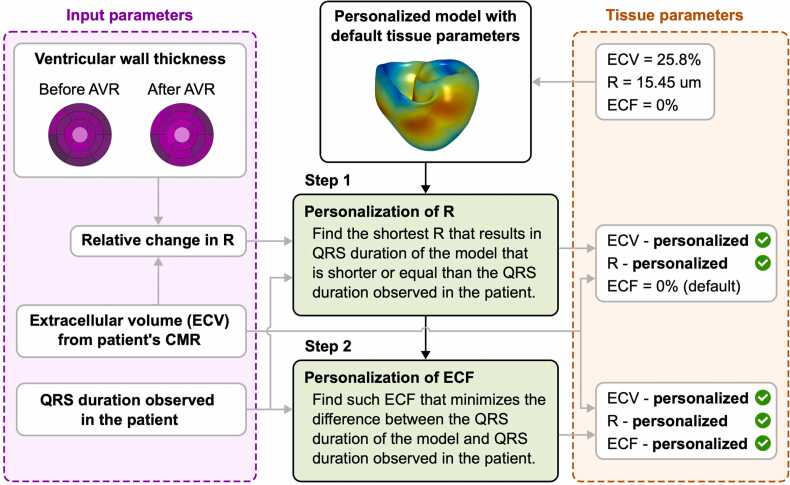
The method for personalizing R and ECF. The method is applied to a personalized biventricular mesh with early activation sites that produce a physiological pattern of ventricular depolarization. In the first step, R is estimated by finding the shortest R that results in a model QRS duration that is shorter than or equal to the QRS duration observed in the patient. ECF is found in the second step by finding the ECF that minimizes the difference between QRS durations of the model and the patient. ECV is calculated from cardiovascular magnetic resonance imaging data and serves as an input parameter. For each patient, the method was applied to the biventricular meshes from before and after AVR. Additional details about the method are provided in the Supplementary Methods. Once the tissue parameters were personalized, simulations of RV apex pacing were performed to measure conduction velocity. *R* cell radius, *ECF* extracellular conductivity factor, *ECV* extracellular volume, *AVR* aortic valve replacement, *RV* right ventricular

To estimate the tissue parameters R and ECF, we developed a method based on fitting the model’s QRS duration to the patient’s QRS duration (Fig. 3). Since ECV is calculated for each patient and time point of the study, it represents a set parameter that does not have to be estimated. The method assumes that the ventricles before and after AVR have the same physiological pattern of ventricular depolarization [23], and that this pattern can be reproduced by stimulation at the EAS [18]. The method is applied to a model with personalized ventricular geometry and a physiological pattern of ventricular depolarization using default tissue parameters (R = 15.45 µm, ECV = 25.8%, ECF = 0%). For simplicity, and to make the method less sensitive to QRS morphology, we approximated the model’s QRS duration as the total activation time of the ventricles [19], [20].

First, we performed simulations to find the shortest R that results in a QRS duration that is shorter than or equal to the QRS duration observed in the patient. R had to be within the range of values observed in AS patients, determined as 9.6–17.8 µm (data by Krayenbuehl et al. [9], mean R observed in AS patients ± 1 standard deviation). In addition, the ratio between the Rs in the pre- and post-AVR models had to be fixed and given by the relative change in R calculated from the average wall thicknesses and ECV values before and after AVR. The Rs before and after AVR were determined iteratively by performing simulations in both models from the same patient. In the second step, we adjusted ECF. If the model’s QRS duration remained shorter than the patient’s QRS duration, the ECF was increased to minimize the difference between the QRS durations. To avoid unnecessary simulations and to reduce the overall computational time, ECF was increased in steps of 5%. Additional details regarding the method are provided in the Supplementary Methods.

### 2.6. Simulation protocol

Each simulation consisted of 1000 ms of no electrical stimulation to allow for internal model parameters to equilibrate, followed by S1 pacing with a cycle length of 600 ms until steady state. For the simulations that were performed to estimate the tissue parameters R and ECV, the models were paced from EAS to mimic a physiological pattern of ventricular depolarization. These simulations were used to obtain QRS duration. In the simulations used for measuring CV, the models were paced from the RV apex.

To save computational time, the simulations were performed as monodomain. Previous studies have shown that there are only minimum differences between monodomain and bidomain simulations of action potential propagation [30]. All simulations were performed using the cardiac electrophysiology simulator CARPentry (https://www.numericor.at) running on the Joliot-Curie TGCC supercomputer (https://www-hpc.cea.fr/en/Joliot-Curie.html).

### 2.7. Data analysis and statistics

LV mass was calculated as the product of LV volume and the specific gravity of the myocardium (1.05 g/mL)[4]. The extracellular matrix volume was calculated as the product of LV volume and ECV. Cell volume was calculated as the product of LV volume and 1-ECV [4]. The LV mass, extracellular matrix volume, and cell volume were indexed to the body surface area. CV was measured in the personalized computational models using two points along the apicobasal axis in the RV septum positioned approximately 2 cm apart (as illustrated in Supplementary Fig. S2). A Wilcoxon matched-pairs signed rank test was used to compare patient characteristics and other paired data. Sex-specific differences were compared with a Mann-Whitney test. The Spearman’s correlation coefficient was used to assess the correlation between variables. Data were presented as the median and interquartile range unless stated otherwise.

## 3. Results

Data from 12 patients with severe AS were used in this study (Table 1). CMR was acquired 2 (1–3) days before AVR and 119 (96–135) days after AVR.

**Table 1:** Patient characteristics

	All patients (n = 12)	Before AVR	After AVR	p-value
Age, y	64 [53–74]			
Male sex, n (%)	6 (50)			
Body surface area, m^2^	1.9 [1.6–2.0]			
EDV, indexed, mL/m^2^		69 [64–86]	63 [58–70]	0.0425
ESV, indexed, mL/m^2^		31[25–45]	26[18–29]	0.0024
Ejection fraction, %		55 [52–62]	62 [53–67]	0.1553
LV mass, indexed, g/m^2^		62 [58–79]	51 [41–60]	0.0005
Extracellular volume (%)		24.2 [20.6–24.8]	28.0 [25.1–29.5]	0.0008
QRS duration, ms		89 [81–99]	88 [79–100]	0.2148

Values are presented as the median and interquartile range

*AVR* aortic valve replacement, *EDV* end-diastolic volume, *ESV* end-systolic volume, *LV* left ventricle

### 3.1. Left ventricular remodeling after AVR

**Fig. 4:**
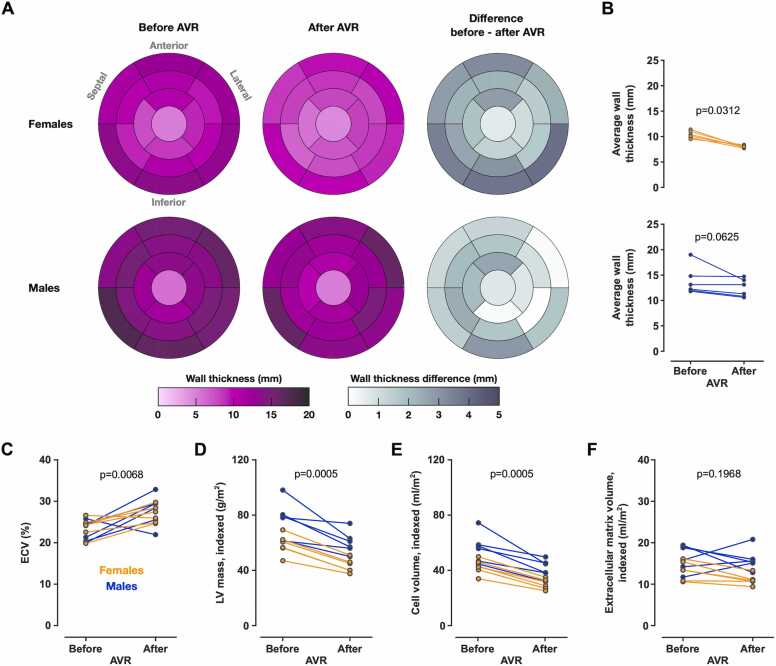
LV remodeling after AVR. (A) Bull’s eye plots depicting LV wall thickness before and after AVR for each AHA segment [26]. The wall thickness before and after AVR is color-coded with the purple scale, and the before-after AVR difference is color-coded with the gray scale. (B) The overall reduction in LV wall thickness was rather uniform in both sexes and was more pronounced in females. (C) AVR was followed by an increase in ECV, (D) a decrease in LV mass, and (E) a decrease in cell volume. (F) Only small changes in the extracellular matrix volume were found. Data from 6 female and 6 male patients are presented. In panel D, data points from 2 female patients overlap (Patient #1: 56.2 and 40 g/m^2^; Patient #2: 56.5 and 40 g/m^2^; before and after AVR, respectively). In panel F, data points from 2 female patients overlap (Patient #2: 13.4 and 10.7 mL/m^2^; Patient #3: 13.4 and 10.9 mL/m^2^; before and after AVR, respectively). *AVR* aortic valve replacement, *LV* left ventricular, *AHA* American Heart Association, *ECV* extracellular volume

A decrease in LV mass was observed after AVR (62 [58–79] g/m^2^ to 51 [41–60] g/m^2^, p = 0.0005) that was associated with a 15% reduction in LV wall thickness (11.6 [10.2–12.9] mm to 9.5 [7.9–12.7] mm, p = 0.001). The wall thickness reduction was more pronounced in females (21%, 10.3 [9.7–11.1] mm before AVR, 8.0 [7.9–8.2] mm after AVR, p = 0.0312) than in males (8%, 12.7 [12.0–15.9] mm before AVR, 12.2 [10.8–14.18] mm after AVR, p = 0.0625, Fig. 4B). Average wall thicknesses for each patient and time point are provided in Supplementary Table S2. The change in wall thickness was non-uniform, with the most pronounced reduction at the LV basal inferior, basal infero-lateral, infero-septal, and anterior regions (Fig. 4A).

Ventricular remodeling was associated with an increase in ECV (24.2 [20.6–24.8] % before AVR, 28.0 [25.1–29.5] % after AVR, p = 0.0008, Fig. 4C) but no sex-specific differences in ECV were found (females: 24.9 [24.2–27.2] %, males: 25.1 [21.5–29.2] %, p = 0.8539). The reduction in LV mass was driven mainly by a reduction in the cell volume (46.3 [42.5–57.2] mL/m^2^ before AVR, 34.0 [29.5–42.1] mL/m^2^ after AVR, p = 0.0005, Fig. 4E). Only small changes in the extracellular matrix volume were found (14.8 [12.1–18.1] mL/m^2^ before AVR, 13.1 [10.8–15.5] mL/m^2^ after AVR, p = 0.1968, Fig. 4F). The cell volume was lower in females than in males (34.5 [29.5–42.8] mL/m^2^ and 46 [39.9–57.2] mL/m^2^, p = 0.0068), and the same observation was made for the extracellular matrix volume (11.0 [10.7–13.4] mL/m^2^ and 15.7 [14.4–19.0] mL/m^2^, p = 0.0013). Only minor changes in the QRS duration were observed between the time points (89.0 [80.5–99.0] ms before AVR 88 [78.5–99.5] ms after AVR, p = 0.2148).

### 3.2. Personalized digital twins of human ventricles

**Fig. 5:**
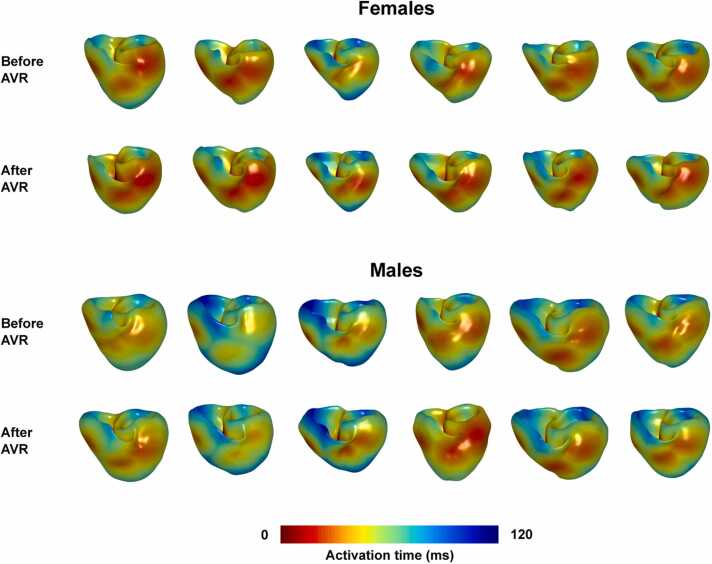
Personalized biventricular models showing the pattern of physiological depolarization. *AVR* aortic valve replacement

**Fig. 6:**
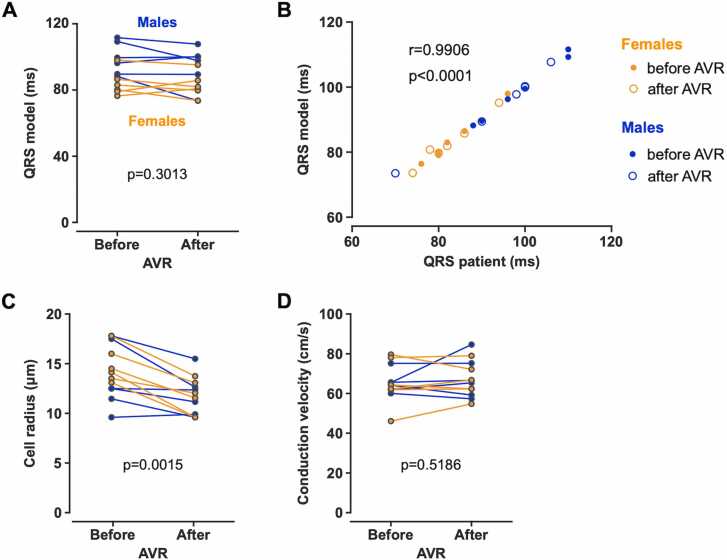
Parameters derived from the personalized digital twins of the human ventricles. (A) Only minor changes in QRS duration were found between the time points. (B) QRS durations of the personalized computational models closely correlated with the QRS durations acquired in the patients. (C) Cell radius was found to be smaller in the post-AVR models than in the pre-AVR models. (D) Post-AVR models had a similar conduction velocity to pre-AVR models. Data from 6 female and 6 male patients are presented. In panel B, data points from two male patients after AVR overlap (blue circles; Patient #1: 100 and 100.25 ms, Patient #2: 100 and 99.8 ms; patient’s QRS durations and QRS durations estimated from the model, respectively). *AVR* aortic valve replacement

For each patient and time point, a personalized digital twin of the ventricles was developed. Detailed information about the biventricular meshes is provided in Supplementary Table S3. A physiological pattern of ventricular depolarization was simulated in each model (Fig. 5) using EAS stimulation (see Supplementary Table S4 for patient-specific EAS settings). There was no difference in the model QRS duration between the time points (88.9 [80.9–99.1] ms before AVR, 87.6 [80.0–99.4] ms after AVR, p = 0.3013, Fig. 6A). Model QRS durations closely correlated with the QRS durations acquired in the patients (p<0.0001, r = 0.9906, Fig. 6B), with the median fitting error not exceeding 0.5 ms (0.49 [0.31–0.98] ms before AVR, 0.33 [0.23–1.57] ms after AVR, p = 0.6221).

### 3.3. Ventricular tissue changes and CV in the digital twins

The R estimated by the computational method was larger before AVR than after AVR (13.8 [12.5–17.1] μm and 11.8 [9.7–13.00] μm, p = 0.0015, Fig. 6C). No sex-specific differences in R were found (females: 13.3 [11.7–14.4] μm; males: 12.4 [10.2–14.8], p = 0.3481). Only small and negligible changes in CV were observed between the models from the two distinct time points (64.3 [61.9–72.8] cm/s before AVR, 66.0 [60.0–74.5] cm/s after AVR, p = 0.5186, Fig. 6D) that showed no effect of reverse ventricular remodeling on CV.

### 3.4. The association between ECV, R, and CV

**Fig. 7:**
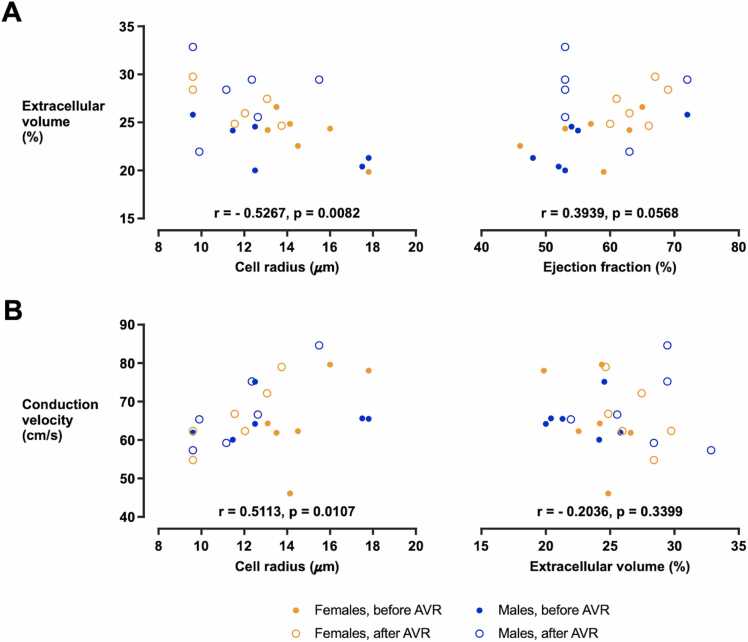
Correlations between the selected clinical and model-derived parameters. (A) A negative association was found between the ECV and R derived from the models, and a borderline insignificant positive association was found between ECV and ejection fraction. (B) A positive association was found between the R and CV derived from the models. No correlation was found between ECV and CV, suggesting that the increase in ECV observed after AVR is not associated with an increase in diffuse fibrosis that would lead to CV slowing. Data from 6 female and 6 male patients are presented. *ECV* extracellular volume, *R* cell radius, *CV* conduction velocity, *AVR* aortic valve replacement

A negative correlation was found between ECV and R (r = −0.5267, p = 0.0082, Fig. 7A) which suggests that the ECV increase observed after AVR was associated with a reversion of cellular hypertrophy. When correlated with clinical parameters, a weak correlation was found between the ECV and ejection fraction at the borderline of statistical significance (r = 0.3939, p = 0.0568, Fig. 7A). No association was found between ECV and end-diastolic volume (r = −0.2762, p = 0.1936, Table 2), nor between ECV and the remaining model parameters (Table 2).

**Table 2:** Correlations between clinical and model-derived parameters

	Extracellular volume	Cell radius	Conduction velocity
Extracellular volume	----	----	----
Cell radius	r = −0.5267# p = 0.0082	----	----
Conduction velocity	r = −0.2036# p = 0.3399	r = 0.5113# p = 0.0107	----
End-diastolic volume	r = −0.2762 p = 0.1936	r = 0.2866 p = 0.1746	r = 0.3280 p = 0.1176
Ejection fraction	r = 0.3939# p = 0.0568	r = −0.3459 p = 0.0978	r = −0.0140 p = 0.9481

The table presents values of the Spearman's correlation coefficient (r) and the respective p-value.

# Graph shown in Fig. 7

CV measured in the computational models was positively correlated with R (r = 0.5113, p = 0.0107, Fig. 7B). This observation agrees with the sensitivity analysis showing that CV is increased in cellular hypertrophy (Supplemental Fig. S4). No association was found between CV and ECV (r = −0.2036, p = 0.3399, Fig. 7B), suggesting that the post-AVR increase in ECV is not associated with an increase in diffuse fibrosis that would cause a slowing of ventricular conduction.

## 4. Discussion

This study presents a new approach for estimating ventricular tissue properties in AS patients. The presented method combines CMR and ECG data to develop personalized digital twins. By applying this method to data from patients with no abnormalities in ventricular conduction and no LGE scar, we provide new insights into the ventricular remodeling that follows AVR. This demonstrates that increased ECV three months after AVR is not associated with decreased ventricular CV, suggesting no substantial increase in diffuse myocardial fibrosis. Our findings indicate that in these patients, increased ECV after AVR appears to be a physiological marker of successful regression of cellular hypertrophy rather than pathological fibrosis, and is likely benign.

### 4.1. Regression of LV mass

Reverse ventricular remodeling that follows AVR is associated with a reduction in LV mass [4]. The LV mass reduction observed in our patients three months after AVR (from 62 [58–79] to 51 [41–60] g/m^2^, −24%) was larger than in the studies that investigated post-AVR remodeling at the 8-week (from 78 ± 18 to 66 ± 15 g/m^2^, −15%) [7] and one-year time points (from 88 ± 26 to 71 ± 19 g/m^2^, −19%) [4]. This comparison reveals that our cohort consists of patients who already had low LV mass prior to AVR, and responded well to the therapy. From this perspective, the cohort does not fully represent the general population of AS patients. A more pronounced regression of LV mass was found in women than in men. This finding is in agreement with previously described sex-specific differences in the regression of LV hypertrophy after AVR [31].

### 4.2. Regression of cellular hypertrophy

The ventricles after AVR showed a regression of cellular hypertrophy. This was identified both as a decrease in cell volume (a CMR-derived parameter) and a shortening of R (a model-derived parameter). The decrease in cell volume was substantial (from 46.3 [42.5–57.2] to 34.0 [29.5–42.1] mL/m^2^, −28%) and considerably contributed to the LV mass reduction. The estimated R in the ventricles before AVR (13.8 [12.5–17.1] μm) was shorter than the values reported in the histological study by Krayenbuehl et al. (15.45 ± 2.35 µm) [9]. The R after AVR (11.8 [9.7–13.0] μm) was slightly larger than the control values reported in the same study (10.6 ± 1.0 µm), offering an interpretation that in some patients, cellular hypertrophy due to AS might not be fully reversed three months after AVR. A comparison of cell diameters estimated from the models and those reported by the histological study is provided in Supplemental Fig. S6. We speculate that the level of cellular hypertrophy in AS patients is nowadays lower than in 1989 when the study was published since therapeutic approaches for AS treatment have evolved and the demographics of AS patients have changed over the past decades.

### 4.3. Post-AVR changes in extracellular space

The ECV increase observed in our cohort three months after AVR (from 24.2 [20.6–24.8] to 28.0 [25.1–29.5] %, +4.8%) was more pronounced than the ECV increases reported at 8 weeks (from 27.4 ± 3.1 to 30.2 ± 2.8%, +2.8%) [7] and one year after AVR (from 28.2 ± 2.9 to 29.9 ± 4.0%, +1.2%) [4]. This is likely because our cohort consists of patients who generally responded well to AVR, as previously stated. The overall low ECV in our patients, both before and after AVR, could have been caused by the strict inclusion criteria that led to the exclusion of patients with LGE scar. The ECVs observed in our patient cohort before AVR were mostly within normal values [2], and the ECVs observed after AVR were at levels that are common before AVR [4], [6], [7], [8].

When extracellular matrix volume was calculated as the product of LV volume and ECV, we observed only a small reduction in the extracellular matrix volume three months after AVR (from 14.8 [12.1–18.1] to 13.1 [10.8–15.5] mL/m^2^, −9%). A similarly small decrease in extracellular matrix volume was reported in AS patients 8 weeks after AVR (from 20.6 ± 5.8 to 19.1 ± 4.9 mL/m^2^, −7.2%) [7]. In contrast, a more pronounced reduction in extracellular matrix volume was reported one year after AVR (from 25 ± 9 to 21 ± 7 mL/m^2^, −16%). Although our patient cohort differs from the general AS population, and despite the limitations associated with this, our observation regarding extracellular matrix volume follows the previously proposed hypothesis that a reversion of the extracellular space after AVR is a gradual process that spans for months [4]. This is further supported by previous histological findings that show a reduction in interstitial fibrosis and fibrous content for several years after AVR [9].

### 4.4. Clinical relevance of increased ECV after AVR

Several studies have demonstrated that ECV measured before AVR can be used as a predictor of mortality [5], [6]. This has also been shown for ECV estimated by computed tomography [32]. We demonstrate in our computational study that an increase in ECV three months after AVR in the patients with no LGE scar is only apparent [7] and not necessarily associated with tissue changes that would lead to a decrease in ventricular CV.

Our findings show that the LV mass reduction observed three months after AVR was caused mainly by a regression in cell volume, while the regression in extracellular matrix volume contributed to the LV mass reduction only marginally. As a consequence, the ratio between extracellular and cellular volume, expressed as ECV, shifted towards the extracellular volume and resulted in an ECV increase. This finding is in line with the recent observations by Bennett et al. [7], and is further supported by our demonstration of a negative correlation between the R and ECV. Based on these observations, we propose that elevated ECV after AVR is merely a footprint of regressed cellular hypertrophy that accompanies reverse ventricular remodeling.

### 4.5. New approach for computational modeling of ventricular tissue

For the purpose of this study, we developed a new method for modeling ventricular tissue in patients with hypertrophy. The method is based on our previous work [10] and uses cutting-edge digital twins of the human ventricles derived from CMR and ECG data. The Rs estimated by our method are very similar to the cell radii reported by a previous histological study (for the comparison see Supplementary Fig. S6) [9]. Additional studies are necessary to verify how closely the estimated Rs correlate with the values obtained by standard histological methods. Nevertheless, the absence of these studies does not hinder our conclusions. Both R and ECF are internal model parameters, and the main finding (i.e., increased ECV three months after AVR is not associated with decreased CV) can be achieved without them. Instead of using parameters with physiological meaning, the tissue personalization could have been performed with a single scaling factor for adjusting tissue conductivity. We scaled the tissue conductivity through R and ECF because utilizing parameters related to actual physiological properties of cardiac tissue can provide more insights into ventricular remodeling than applying an approach based on a single scaling factor that does not have any physiological meaning.

The integration of both CMR and ECG data into a personalized computational model allowed for a non-invasive estimation of ventricular CV. This approach goes beyond the information provided by ECG data alone, and highlights a potentially promising application for digital twins of the human ventricles [33]. However, further studies are required to validate its accuracy, especially when a general pattern of ventricular depolarization is mimicked by stimulation from EAS [18].

### 4.6. Applicability of the modeling approach in other cardiac diseases

The method presented in this study can be used for modeling ventricles in patients with diseases other than AS. However, several prerequisites have to be fulfilled. First, there should be a difference in the degree of ventricular hypertrophy between the time points. Second, the method can be applied only on ventricles in which a physiological pattern of ventricular depolarization can be assumed. The presence of a scar, focal fibrosis, or bundle branch block that can alter the physiological pattern of ventricular depolarization prevent its use. Third, the method assumes no substantial changes in intracellular conductivity due to a different distribution of gap junctions between the time points. Therefore, utilization of the method in patients for whom such an assumption cannot be made should be done with caution.

It has been shown that in AS patients with compensated LV hypertrophy (ejection fraction >50%) increased lateralization of gap junctions can be observed, even though the amount of gap junctions per cell area is the same as in the cardiomyocytes of control patients [34]. Although this finding might imply an increased (intra)cellular conductivity in the lateral directions in the patients included in this study, we did not make such an adjustment in our digital twins. The sensitivity analysis for transversal and normal intracellular conductivity (Supplementary Fig. S5) revealed that simultaneous scaling of intracellular conductivity in these directions has only a minor effect on CV and QRS duration. We therefore decided not to take these changes into account.

### 4.7. Translational perspective

The results presented in this study suggest that increased ECV after AVR is not associated with increased ventricular fibrosis, and is therefore benign. Furthermore, our findings offer a feasible translational perspective that the early post-AVR increase in ECV might be a marker of healthy reverse ventricular remodeling, as well as an indication of a positive response to AVR. In line with such reasoning, one can speculate that the patients with a small post-AVR ECV increase might have a suboptimal response to AVR and could therefore require closer monitoring. Additional studies are, of course, necessary to validate these hypotheses and to test them in cohorts that are more representative of general AS patient populations.

## 5. Limitations

Low sample size and strict inclusion criteria represent major limitations of this study. Due to the exclusion criteria, the studied cohort does not fully reflect the general population of AS patients. Given the low ECV values, the cohort more likely represents patients with a low risk for cardiovascular mortality [5], [6]. Translating our findings to larger patient cohorts, especially to those with LGE CMR scar, should therefore be done with caution. The low sample size was mainly determined by the availability of the patients who fitted the inclusion criteria, and also by the need for developing two computational meshes for each patient which can be a time-consuming task. The sample size was also limited by the overall computational demand of the method, which required several simulations to be performed in each model in order to estimate the tissue parameters. Another limitation is the availability of only one post-AVR time point three months after AVR. A later time point, such as one year after AVR, would allow for further insight into the reverse ventricular remodeling, direct comparisons with previous studies [4], as well as to strengthen the study's conclusions.

Our finding that CV is not altered by post-AVR ventricular remodeling is derived from measurements in computational models of the human ventricles, not from direct CV measurements in patients. Although the absence of invasive CV measurements can be seen as a limitation, such measurements would be ethically complicated and risky, especially in the post-AVR patients. The same holds true for the model-derived parameters R and ECF that estimate the degree of cellular hypertrophy and amount of diffuse fibrosis, respectively. A validation study is necessary to characterize their relationship with histology-derived parameters.

The computational method presented in this study is based on several assumptions that need to be considered. First, we assume a generic healthy pattern of ventricular depolarization [23] that can be mimicked by pacing from EAS [18]. However, this pattern might vary considerably, even in healthy humans, as demonstrated by a recent ECG imaging study [35]. Incorporating patient-specific ventricular depolarization patterns into our method could possibly lead to a more precise CV estimation, as well as to a less strict inclusion criteria. Second, fitting the model’s CV to generate total activation times that approximate QRS duration can lead to a slightly faster CV than when fitting the model to only QRS duration. [19]. Although the CVs reported in our study can be somewhat overestimated, it is unlikely that this aspect affects our findings or conclusions because the QRS duration was obtained in the same way for both time points. Finally, our method for estimating R and ECF does not consider any changes in sodium channels, gap junctions, or other active membrane properties between the time points. The method can be extended to incorporate these changes if relevant data become available.

The change in ECV reported in this study may be slightly overestimated due to different rates of cell-interstitial water exchange in the ventricles at different levels of cellular hypertrophy [36]. In the ventricles before AVR, the cellular hypertrophy slightly limits the water exchange, leading to underestimation of ECV. This is consistent with the ECV estimates from computed tomography which are not sensitive to the water exchange and are slightly larger than the ECV values reported in this study [32]. In contrast, the reduced or absent cellular hypertrophy after AVR facilitates the water exchange, leading to a more accurate estimation of ECV. Nevertheless, this phenomenon has only a marginal effect on the findings reported in our study.

## 6. Conclusion

We present a new computational approach for estimating the degree of cellular hypertrophy in hypertrophic ventricles. Using this method, we demonstrate that increased ECV three months after AVR is not associated with decreased ventricular conduction velocity, which suggests a negligible change in diffuse myocardial fibrosis. Our findings indicate that increased ECV after AVR in patients with no LGE CMR scar and a physiological pattern of ventricular depolarization can be considered merely as a footprint of successful regression of cellular hypertrophy that is likely benign. Additional studies are warranted to validate our findings against histology or another method that directly quantifies the amount of diffuse cardiac fibrosis.

## Funding

This research was funded by the French National Research Agency (ANR) grant ANR-10-IAHU-04, the European Union’s Horizon 2020 research and innovation program under the ERA-NET co-fund action No. 680969 (ERA-CVD: SICVALVES) and by Fondation Lefoulon-Delalande (VS). It was co-funded by the Austrian Science Fund (FWF), Grant I 4652-B (to CMA), and by the French National Research Agency grant ANR-19-ECVD-0006 (to JDB). The project was provided with high performance computing and storage resources by Grand Equipment National de Calcul Intensif (GENCI) at Trés Grand Centre de Calcul (TGCC) thanks to the grant 2024-A0160310517 on the supercomputer Joliot Curie's ROME partition.

## Author contributions

**Vladimír Sobota:** Writing – review & editing, writing – original draft, visualization, validation, methodology, investigation, formal analysis, data curation, conceptualization. **Christoph M. Augustin:** Writing – review & editing, resources, funding acquisition, data curation, conceptualization. **Gernot Plank:** Writing – review & editing, supervision, software. **Edward J. Vigmond:** Writing – review & editing, supervision, software, resources. **Sarah Nordmeyer:** Writing – review & editing, methodology, funding acquisition, data curation, conceptualization. **Jason D. Bayer:** Writing – review & editing, writing – original draft, supervision, software, resources, methodology, funding acquisition, conceptualization.

## Declaration of competing interest

The authors declare that they have no known competing financial interests or personal relationships that could have appeared to influence the work reported in this paper.

## Data Availability

The data underlying this article will be shared on reasonable request to the corresponding author.

## Footnotes

Appendix A Supplementary data associated with this article can be found in the online version at doi:10.1016/j.jocmr.2025.101936.
