# Supplementary material for: Increased extracellular volume after aortic valve replacement: A footprint of reverse ventricular remodeling that does not affect conduction velocity
Source: J Cardiovasc Magn Reson. 2025 Aug 6;27(2):101936. doi: 10.1016/j.jocmr.2025.101936 (PMC12673024; doi:10.1016/j.jocmr.2025.101936)
Supplement: Supplementary file 1 — Supplementary material [file mmc1.pdf]

Increased extracellular volume after aortic valve replacement: A footprint of reverse ventricular remodeling that does not affect conduction velocity

Supplementary Material

Vladimír Sobota[1,2], Christoph M. Augustin[3,4], Gernot Plank[3,4], Edward J. Vigmond[1,2], Sarah Nordmeyer[5], Jason D. Bayer[1,2]

[1]IHU Liryc, Electrophysiology and Heart Modeling Institute, Fondation Bordeaux Université, Bordeaux, France
[2]University of Bordeaux, Institut de Mathématiques de Bordeaux, UMR 5251, Talence, France
[3]Medical University of Graz, Graz, Austria
[4]BioTechMed-Graz, Graz, Austria
[5]Department of Diagnostic and Interventional Radiology, Tübingen University Hospital, University of Tübingen, Tübingen, Germany
[6]Institute of Computer-Assisted Cardiovascular Medicine, German Heart Center Charité, Berlin, Germany

1 Supplementary Methods

1.1 Creating personalized digital twins of human ventricles

Personalized computational models of the human ventricles were created following the general framework for the generation of digitals twins, as described by Gillette et al. [1]. Balanced steady-state free precession (bSSFP) cardiovascular magnetic resonance (CMR) imaging data were segmented using the semiautomatic segmentation protocol in the MUSIC software [2]. The segmentations were then manually corrected in Seg3D (http://www.seg3d.org) and converted to surface meshes using Meshtool (https://bitbucket.org/aneic/meshtool/src/master/). NumeriCor Studio (https://www.numericor.at) was used to generate tetrahedral volumetric meshes, aiming for an average mesh element edge resolution of 475 μm. Properties of all biventricular meshes (number of nodes, number of elements, average edge length) are provided in Table S3. Myocardial fibers were assigned to each mesh element using an established rule-based approach [3]. Ventricular coordinates were determined [4] to define endocardial, mid-myocardial, and epicardial regions. These regions allowed setting up specific tissue conductivities in the endocardial, mid-myocardial, and epicardial layer of the ventricular wall (Table S1). Each model was assigned with five early activations sites (EAS) to allow for simulations that mimicked physiological pattern of ventricular depolarization [1]. The EAS locations were manually adjusted where necessary. The final EAS locations for each model, described by universal ventricular coordinates, can be found in Table S4. In addition, a stimulation site at the endocardial side of the right ventricular (RV) apex was defined to allow for simulations of RV apical pacing. Human cellular electrophysiology was simulated by the ten Tusscher ventricular myocyte model [5].

1.2 Estimation of relative cell radius change

Our simulations assume a tissue model with cylindrical cardiac cells that are parallel to each other (Figure 2A) with a radius R, positioned in an extracellular space of width w (Figure S1). The R and w from the time point

before aortic valve replacement (AVR) will be referred to as R_{before} and w_{before}, respectively, and the values after AVR will be referred to as R_{after} and w_{after}, respectively. The thickness of ventricular wall before AVR (W_{before}) and after AVR (W_{after}) can be approximated as

$$W_{\text{before}} = N_{\text{before}} \cdot w_{\text{before}} \,, \tag{1}$$

$$W_{\text{after}} = N_{\text{after}} \cdot w_{\text{after}} \,, \tag{2}$$

where N_{before} and N_{after} are the numbers of myocytes across the ventricular wall before and after AVR, respectively. Assuming that the reverse myocardial remodeling that follows AVR is associated with a negligible change in the number of cardiomyocytes, the number of myocytes in the ventricles before and after AVR can be considered the same, hence $N_{\text{before}} \approx N_{\text{after}}$. The relative change in myocardial wall thickness before and after AVR is then proportional to the ratio

$$\frac{W_{\text{after}}}{W_{\text{before}}} = \frac{w_{\text{after}}}{w_{\text{before}}} \,. \tag{3}$$

Applying the simple geometry of our tissue model (cylindrical cardiac cells in extracellular space), the fractions of intracellular space before AVR (ICV_{before}) and after AVR (ICV_{after}) can be calculated as

$$ICV_{\text{before}} = \frac{\pi R^2_{\text{before}}}{w^2_{\text{before}}} \,, \tag{4}$$

$$ICV_{\text{after}} = \frac{\pi R^2_{\text{after}}}{w^2_{\text{after}}} \,. \tag{5}$$

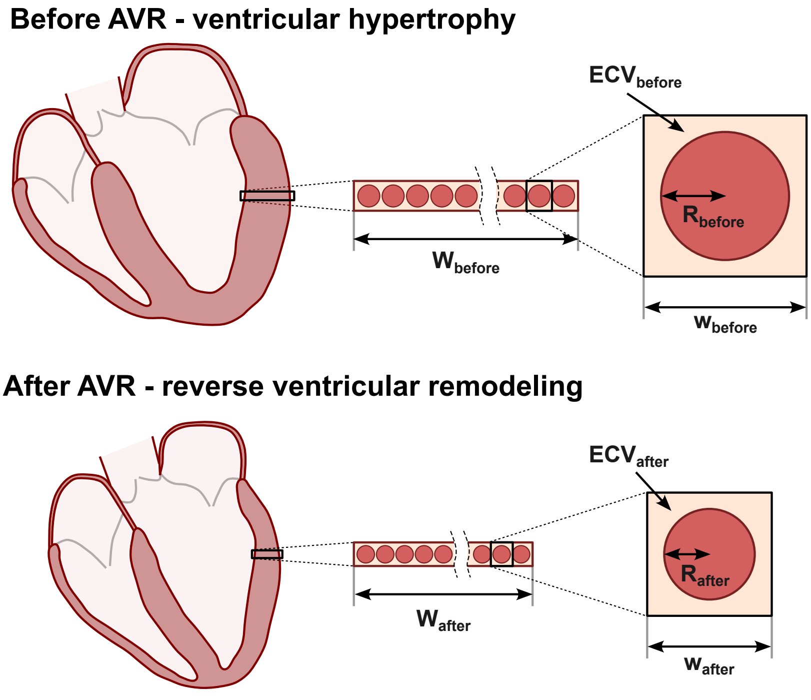

Figure S1: Parameters for estimating relative change in cell radius.

Combining the equations (3), (4), and (5), the relative change in wall thickness can be expressed as

$$\frac{W_{\text{after}}}{W_{\text{before}}} = \frac{R_{\text{after}}}{R_{\text{before}}} \sqrt{\frac{ICV_{\text{before}}}{ICV_{\text{after}}}} \,. \tag{6}$$

Given that extracellular volume (ECV) and ICV are related as $ICV = 1 - ECV$, the relative cell radius change k

can be estimated using the wall thicknesses W_{before}, W_{after}, and ECV acquired before AVR (ECV_{before}) and after AVR (ECV_{after}):

$$k = \frac{R_{\text{after}}}{R_{\text{before}}} = \frac{W_{\text{after}}}{W_{\text{before}}}\sqrt{\frac{1 - ECV_{\text{after}}}{1 - ECV_{\text{before}}}} \,. \tag{7}$$

This expression allows to estimate the relative cell radius change k using the parameters that can be easily obtained from 3-dimensional computational models (average wall thicknesses W_{before}, W_{after}) and from CMR (extracellular volumes ECV_{before}, ECV_{after}).

To obtain k that is representative for the whole biventricular model, we calculated W_{before} and W_{after} as average wall thicknesses from the 17 American Heart Association segments [6] obtained from the models from before and after AVR, respectively. The average wall thicknesses before and after AVR (W_{before}, W_{after}), and ECV before and after AVR (ECV_{before}, ECV_{after}), together with the relative cell radius change k calculated for each patient, are presented in Table S2.

1.3 Adjustment of intracellular and extracellular conductivities

To adjust intracellular and extracellular conductivities so they reflect the changes in R and ECV caused by reversed ventricular remodeling that follows AVR, we applied a previously described approach [7], briefly summarized in the text below. As in the calculation of the relative change in cell radius, we are assuming a tissue model with cylindrical cells (Figure S1). In this model, the ratio of membrane area to tissue volume (β) can be calculated as

$$\beta = (1 - ECV)\frac{2}{R} \,. \tag{8}$$

Considering the default β of the tissue model to be $\beta_d = 0.14\ \mu\text{m}^{-1}$ and the cell radius in healthy human hearts $R_{\text{healthy}} = 10.6\ \mu\text{m}$ [8], the default ECV of the tissue model (ECV_d) was determined from equation (8) as

$$ECV_d = 1 - \frac{\beta_d \cdot R_{\text{healthy}}}{2} = 1 - \frac{0.14 \cdot 10.6}{2} = 0.258 \approx 25.8\,\%. \tag{9}$$

This value falls within the range of ECV values reported in healthy human volunteers ($26.5 \pm 1.3\,\%$ [9]). To scale the extracellular conductivities, we calculated a scaling coefficient (S_e) as

$$S_e = (1 - ECF)\frac{ECV}{ECV_d} \,, \tag{10}$$

where ECF represents the proportional reduction of extracellular conductivity due to diffuse fibrosis ("extracellular conductivity factor", ECF). The scaling coefficient for intracellular conductivites (S_i) was then calculated as

$$S_i = \frac{1 - ECV}{1 - ECV_d} \,. \tag{11}$$

Bidomain conductivities for longitudinal ($\sigma_{i,l}$, $\sigma_{e,l}$), transversal ($\sigma_{i,t}$, $\sigma_{e,t}$) and normal ($\sigma_{i,n}$, $\sigma_{e,n}$) direction were obtained by multiplying the default conductivities (Table S1)[4] by the respective scaling coefficient [7]. For example, for a tissue with parameters: $ECV = 26.6\,\%$, $R = 13.5\ \mu\text{m}$ and $ECF = 45\,\%$, the ratio of membrane area to tissue volume β and the scaling coefficients S_e and S_i were calculated as

$$\begin{aligned}
\beta &= (1 - ECV)\frac{2}{R} = (1 - 0.266)\frac{2}{13.5} = 0.1087\,\mu\text{m}^{-1}, \\
S_e &= (1 - ECF)\frac{ECV}{ECV_d} = (1 - 0.45)\frac{0.266}{0.258} = 0.567, \\
S_i &= \frac{1 - ECV}{1 - ECV_d} = \frac{1 - 0.266}{1 - 0.258} = 0.989.
\end{aligned}$$

Table S1: Default tissue model parameters.

Parameter	Endocardium	Mid-myocardium	epicardium
Extracellular volume ECV (%)	25.8	25.8	25.8
Cell radius R (μm)	10.6	10.6	10.6
Cell surface to volume ratio β (μm^{-1})	0.14	0.14	0.14
Extracellular conductivity factor ECF (%)	0	0	0
Longitudinal direction			
Extracellular conductivity $\sigma_{e,l}$ (S/m)	0.50	0.50	0.50
Intracellular conductivity $\sigma_{i,l}$ (S/m)	0.50	0.50	0.50
Transversal direction			
Extracellular conductivity $\sigma_{e,t}$ (S/m)	0.20	0.20	0.20
Intracellular conductivity $\sigma_{i,t}$ (S/m)	0.16	0.13	0.10
Normal direction			
Extracellular conductivity $\sigma_{e,n}$ (S/m)	0.20	0.20	0.20
Intracellular conductivity $\sigma_{i,n}$ (S/m)	0.04	0.04	0.04

When setting up early activation sites (EAS), the tissue parameters were set to $ECV = 25.8$ %, $R = 15.45$ μm, and $ECF = 0$ % in all models. This setting is based on the default tissue parameters presented in Table S1 but uses R reported for ventricles of patients with aortic stenosis [8]. The cell surface to volume ratio was adjusted according to equation (8), $\beta = 0.0961$ μm^{-1}.

1.4 Method for personalization of tissue parameters

In this study, we developed and applied a method that estimates the tissue parameters R and ECF using personalized digital twins of human ventricles from before and after AVR. The method is based on the following assumptions:

- The ventricles before and after AVR have the same physiological pattern of ventricular depolarization. Since AVR is associated with an occurrence of bundle branch block (BBB) [10], only patients with QRS$\leq$110 ms both before and after AVR were included. Such QRS threshold excludes patients who can possibly have BBB. In addition, patients with a scar visible on LGE CMR were excluded. Applying these criteria, we assumed a physiological pattern of ventricular depolarization [11] in the patients included in the study. This pattern of physiological depolarization was reproduced by stimulation from EAS [1].

- Once the personalized biventricular models are developed and EAS assigned, the QRS duration of each model depends only on the tissue parameters ECV, R, and ECF. Hence, $QRS_{\text{model,before}}$ is a function of ECV_{before}, R_{before}, and ECF_{before}, and $QRS_{\text{model,after}}$ is a function of ECV_{after}, R_{after}, and ECF_{after}, where $QRS_{\text{model,before}}$ and $QRS_{\text{model,after}}$ are QRS durations obtained from the models before and after AVR, respectively, ECV_{before} and ECV_{after} are extracellular volumes acquired before and after AVR, respectively, and ECF_{before} and ECF_{after} are extracellular conductivity factors before and after AVR, respectively.

  Since ECV_{before} and ECV_{after} were obtained from LGE CMR for each patient and time point, they represent set parameters. The parameters that have to be estimated are therefore R_{before}, R_{after}, ECF_{before}, and ECF_{after}.

- The relative change in R caused by reverse ventricular remodeling can be estimated from the changes in ventricular wall thickness W and ECV, as described in equation (7).

- R should not be shorter than 9.6 μm and should not exceed 17.8 μm. The values are based on a histological study in AS patients by Krayenbuehl et al.[8]. The upper limit was obtained as the mean R in AS patients

before AVR $+1$ standard deviation (SD). The lower limit was obtained as the mean R in control patients -1 SD.

The method for personalization of tissue parameters consists of two steps: personalization of R, and subsequent personalization of ECF (as illustrated in Figure 3):

1. **For each patient, find R_{before} and R_{after}.**

   R_{before} and R_{after} are related as $R_{\text{after}} = kR_{\text{before}}$, where k is the relative change in cell radius defined in equation (7). Both R_{before} and R_{after} should be within the range [9.6; 17.8] μm. ECF_{before} and ECF_{after} are both set to 0 at this point to allow later adjustment of ECF due to the presence of diffuse fibrosis.

   We attempt to find the pair of the shortest R_{before} and R_{after} that fit the criteria described above and yield shorter QRS durations in the models than observed in patients, both before and after AVR ($QRS_{\text{patient,before}}$, $QRS_{\text{patient,after}}$):

$$R_{\text{before}} = \arg\min_{R} \left(QRS_{\text{model,before}}(ECV_{\text{before}}, R, ECF_{\text{before}} = 0) \leq QRS_{\text{patient,before}} \right. \\ \left. \wedge\; QRS_{\text{model,after}}(ECV_{\text{after}}, kR, ECF_{\text{after}} = 0) \leq QRS_{\text{patient,after}}\right)$$

   If no such R_{before} or R_{after} could be found, R_{before} was set to 17.8 μm and $R_{\text{after}} = kR_{\text{before}}$.

2. **For each patient, find personalized extracellular conductivity factors ECF_{before} and ECF_{after}.**

   Both ECF_{before} and ECF_{after} have values in the range [0;1] and should minimize the difference between the QRS obtained in the model ($QRS_{\text{model,before}}$, $QRS_{\text{model,after}}$) and QRS observed in the patient ($QRS_{\text{patient,before}}$, $QRS_{\text{patient,after}}$).

$$\begin{aligned} ECF_{\text{before}} &= \arg\min_{ECF} \left(QRS_{\text{model,before}}(ECV_{\text{before}}, R_{\text{before}}, ECF) - QRS_{\text{patient,before}}\right) \\ ECF_{\text{after}} &= \arg\min_{ECF} \left(QRS_{\text{model,after}}(ECV_{\text{after}}, R_{\text{after}}, ECF) - QRS_{\text{patient,after}}\right) \end{aligned}$$

   The parameters ECF_{before} and ECF_{after} are set independently on each other.

1.5 Conduction velocity measurements

Conduction velocity (CV) measurements were performed in the personalized computational models using two points at the right ventricular septum positioned 2 cm apart (Figure S2). CV was calculated as follows:

$$CV = \frac{|P_1P_2|}{AT_2 - AT_1}\,, \tag{12}$$

where $|P_1P_2|$ is the distance between the points P_1 and P_2 ($\approx$ 2 cm), and AT_1 and AT_2 are the activation times (AT) at the points P_1 and P_2, respectively. The measurements were performed during right ventricular apex pacing with a cycle length of 600 ms.

1.6 Sensitivity analysis

To assess the effect of R, ECV, and ECF on QRS duration, a sensitivity analysis was performed in a representative female ($ECV = 27.45\,\%$, $R = 13.06$ μm, $ECF = 0\,\%$) and male ($ECV = 25.55\,\%$, $R = 12.63\,\mu$m, $ECF = 0\,\%$) post-AVR biventricular meshes. To save computational resources, the effect on CV was evaluated in a tissue slab $9\times1\times1$ cm, incorporated with myocardial fibers and containing endocardial, mid-myocardial, and epicardial layers [12]. Value of each tissue parameter was varied (R: $10-17\,\mu$m; ECV: $20-34\,\%$; ECF: $0-70\,\%$) while keeping the remaining parameters constant. In addition to the effect of tissue parameters, sensitivity analysis was

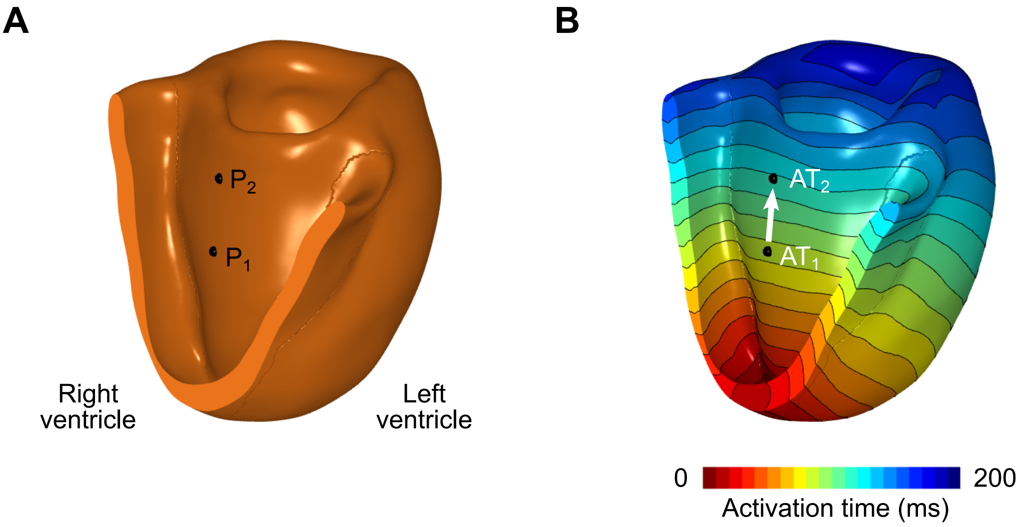

Figure S2: Conduction velocity (CV) measurements. (A) CV was measured in the personalized computational models using two points (P_1 and P_2) at the right ventricular septum positioned 2 cm apart. **(B)** The model was paced from the right ventricular apex, as illustrated by the isochrones. The activation times AT_1 and AT_2 obtained at the points P_1 and P_2, respectively, were used for CV calculation, as described in equation 12. The white arrow indicates the direction of the depolarization wave front between the points P_1 and P_2.

performed for intracellular conductivity (σ_i). To assess directional effects of σ_i, it was scaled in 3 different ways: (a) in longitudinal direction only ($\sigma_{i,l}$), (b) simultaneously in transversal ($\sigma_{i,t}$) and normal ($\sigma_{i,n}$) directions (these directions are both perpendicular to the longitudinal direction), and (c) simultaneously in longitudinal, transversal and normal directions (overall change of σ_i). For each of them, the scaling in a range from 0.5 (50 % decrease) to 1.5 (50 % increase) was applied, using values of $\sigma_{i,l}$, $\sigma_{i,t}$, and $\sigma_{i,n}$ from Table S1 as default.

The biventricular models were stimulated from EAS using the identical stimulation protocol as used in the study, and QRS duration was obtained as the difference between the earliest and the latest activation times. To save computational resources, the effect of σ_i on QRS duration was assessed only in the female biventricular mesh. The tissue model was paced from the lateral side, using the identical stimulation protocol as applied in the biventricular meshes for CV measurement. CV was obtained using two points at the endocardial side of the model positioned 7 cm apart.

2 Supplementary Results

2.1 Wall thickness, ECV, and relative change in cell radius

Average wall thicknesses before and after AVR (W_{before}, W_{after}), ECV before and after AVR (ECV_{before}, ECV_{after}), together with the relative cell radius change k calculated for each patient are presented in Table S2.

2.2 Properties of biventricular meshes

Properties of all biventricular meshes that were generated for this study are presented in Table S3. For each mesh, the table indicates the number of nodes, tetrahedral elements, and the average edge length.

2.3 Locations of early activation sites

Table S4 presents universal ventricular coordinates of early activation sites (EAS) that were used to simulate physiological pattern of ventricular depolarization. According to Bayer et al., z represents the apicobasal, ρ transmural,

Table S2: Wall thicknesses before (W_{before}) and after (W_{after}) aortic valve replacement (AVR), extracellular volume before and after AVR (ECV_{before}, ECV_{after}, respectively), together with relative cell radius change k calculated for each patient. The wall thicknesses represent the average over the 17 American Heart Association segments [6] and are expressed as mean ± standard deviation.

	Wall thickness			Extracellular volume		Cell radius change k (%)
	W_{before} (mm)	W_{after} (mm)	$\frac{W_{\text{after}}}{W_{\text{before}}}$ (%)	ECV_{before} (%)	ECV_{after} (%)	
**Females**						
#1	10.4 ± 2.2	7.9 ± 1.2	78.4	24.4	27.5	76.8
#2	9.8 ± 2.9	7.7 ± 2.1	79.6	19.9	24.7	77.2
#3	11.4 ± 2.5	7.9 ± 1.8	69.6	24.9	28.4	67.9
#4	9.5 ± 3.1	8.4 ± 2.4	88.7	26.6	26.0	89.1
#5	11.0 ± 2.6	8.1 ± 1.2	76.2	24.2	29.8	73.3
#6	10.1 ± 2.6	8.0 ± 1.8	80.8	22.6	24.9	79.6
**Males**						
#1	19.0 ± 4.4	14.0 ± 3.9	74.6	20.4	25.6	72.2
#2	14.8 ± 2.5	14.7 ± 2.3	100.5	25.8	22.0	103.1
#3	12.0 ± 2.8	10.8 ± 3.0	89.0	24.2	32.9	83.8
#4	11.8 ± 3.1	10.6 ± 3.2	91.9	21.3	29.5	87.0
#5	13.1 ± 3.7	13.1 ± 3.5	102.2	24.6	29.5	98.8
#6	12.2 ± 2.7	11.3 ± 2.3	94.3	20.0	28.4	89.2

Table S3: Properties of biventricular meshes used in the study.

**Females**	#1	#2	#3	#4	#5	#6
Before AVR						
Nodes	3,998,001	2,729,662	2,731,075	2,577,800	2,850,622	3,140,560
Elements	23,211,055	15,732,115	15,914,253	14,867,032	16,616,171	18,186,446
Edge length, μm	471 ± 82	478 ± 86	472 ± 82	478 ± 87	480 ± 84	478 ± 85
After AVR						
Nodes	2,606,711	2,542,841	2,153,435	2,428,715	2,529,207	2,413,812
Elements	14,962,243	14,450,102	12,308,061	13,874,402	14,521,598	13,811,907
Edge length, μm	478 ± 85	472 ± 86	471 ± 87	479 ± 89	476 ± 84	471 ± 85
**Males**	#1	#2	#3	#4	#5	#6
Before AVR						
Nodes	6,049,789	6,057,772	3,674,881	3,948,256	5,301,499	4,066,117
Elements	36,022,489	34,617,509	21,349,818	23,104,129	31,037,426	23,796,361
Edge length, μm	473 ± 81	477 ± 99	476 ± 83	478 ± 84	473 ± 84	473 ± 83
After AVR						
Nodes	4,922,839	4,767,989	3,356,336	3,428,636	5,289,678	3,648,659
Elements	28,893,405	26,565,552	19,392,757	19,853,312	30,963,969	20,004,090
Edge length, μm	475 ± 83	475 ± 92	470 ± 85	474 ± 86	474 ± 84	479 ± 104

ϕ rotational, and v ventricular coordinate [3]. Optimal EAS coordinates reported by Gillette et al. [1] are shown for comparison. The same coordinates were used for EAS in both models from the same patient. Only in female patient #4, the ϕ coordinate of the moderator band was set to 0.21 in the pre-AVR model and -0.25 in the post-AVR model. This value is denoted in Table S4 with *.

Table S4: Locations of early activation sites.

	Gillette et al. [1]	Females						Males					
		#1	#2	#3	#4	#5	#6	#1	#2	#3	#4	#5	#6
**LV**													
**Septal**													
z	0.61	0.61	0.61	0.61	0.61	0.51	0.61	0.61	0.51	0.61	0.61	0.51	0.51
ρ	0	0	0	0	0	0	0	0	0	0	0	0	0
ϕ	0.73	0.73	0.83	0.73	0.73	0.73	0.73	0.73	0.73	0.73	0.73	0.73	0.73
v	-1	-1	-1	-1	-1	-1	-1	-1	-1	-1	-1	-1	-1
**Posterior**													
z	0.47	0.47	0.52	0.47	0.47	0.47	0.47	0.47	0.47	0.47	0.52	0.47	0.47
ρ	0	0	0	0	0	0	0	0	0	0	0	0	0
ϕ	-1.36	-1.36	-1.36	-1.36	-1.36	-1.36	-1.36	-1.36	-1.36	-1.36	-1.36	-1.36	-1.36
v	-1	-1	-1	-1	-1	-1	-1	-1	-1	-1	-1	-1	-1
**Anterior**													
z	0.82	0.82	0.72	0.72	0.77	0.72	0.77	0.72	0.72	0.77	0.77	0.72	0.72
ρ	0	0	0	0	0	0	0	0	0	0	0	0	0
ϕ	1.94	1.94	2.04	1.94	1.94	1.94	1.94	1.94	1.94	1.94	2.1	1.94	1.94
v	-1	-1	-1	-1	-1	-1	-1	-1	-1	-1	-1	-1	-1
**RV**													
**Septal**													
z	0.73	0.73	0.73	0.73	0.73	0.73	0.73	0.73	0.73	0.73	0.73	0.73	0.73
ρ	1	1	1	1	1	1	1	1	1	1	1	1	1
ϕ	-0.04	-0.04	-0.04	-0.04	-0.04	-0.04	-0.04	-0.04	-0.04	-0.04	-0.04	-0.04	-0.04
v	-1	-1	-1	-1	-1	-1	-1	-1	-1	-1	-1	-1	-1
**Moderator band**													
z	0.63	0.63	0.63	0.63	0.63	0.63	0.63	0.63	0.63	0.63	0.63	0.63	0.63
ρ	0.11	0.11	0.11	0.11	0.11	0.11	0.11	0.11	0.11	0.11	0.11	0.11	0.11
ϕ	0.21	0.1	0.1	0.21	0.21*	0.21	0.21	0.21	0.21	0.21	0.1	0.21	0.21
v	1	1	1	1	1	1	1	1	1	1	1	1	1

2.4 Extracellular conductivity factor

Extracellular conductivity factor (ECF) contains both the information about extracellular tissue conductivity, and residuals from QRS fitting. Figure S3A shows that overall, there was no trend in the ECF change from pre-AVR to post-AVR. No association between ECV and ECF was found (Figure S3B), further supporting the study findings, and showing that a particular ECV level can represent different levels of tissue fibrosis. A negative correlation was found between ECF and conduction velocity (Figure S3C), which is in agreement with the sensitivity analysis presented in Figure S4. We did not observe any association between ECF and R, neither between ECF and ejection fraction (Figure S3C).

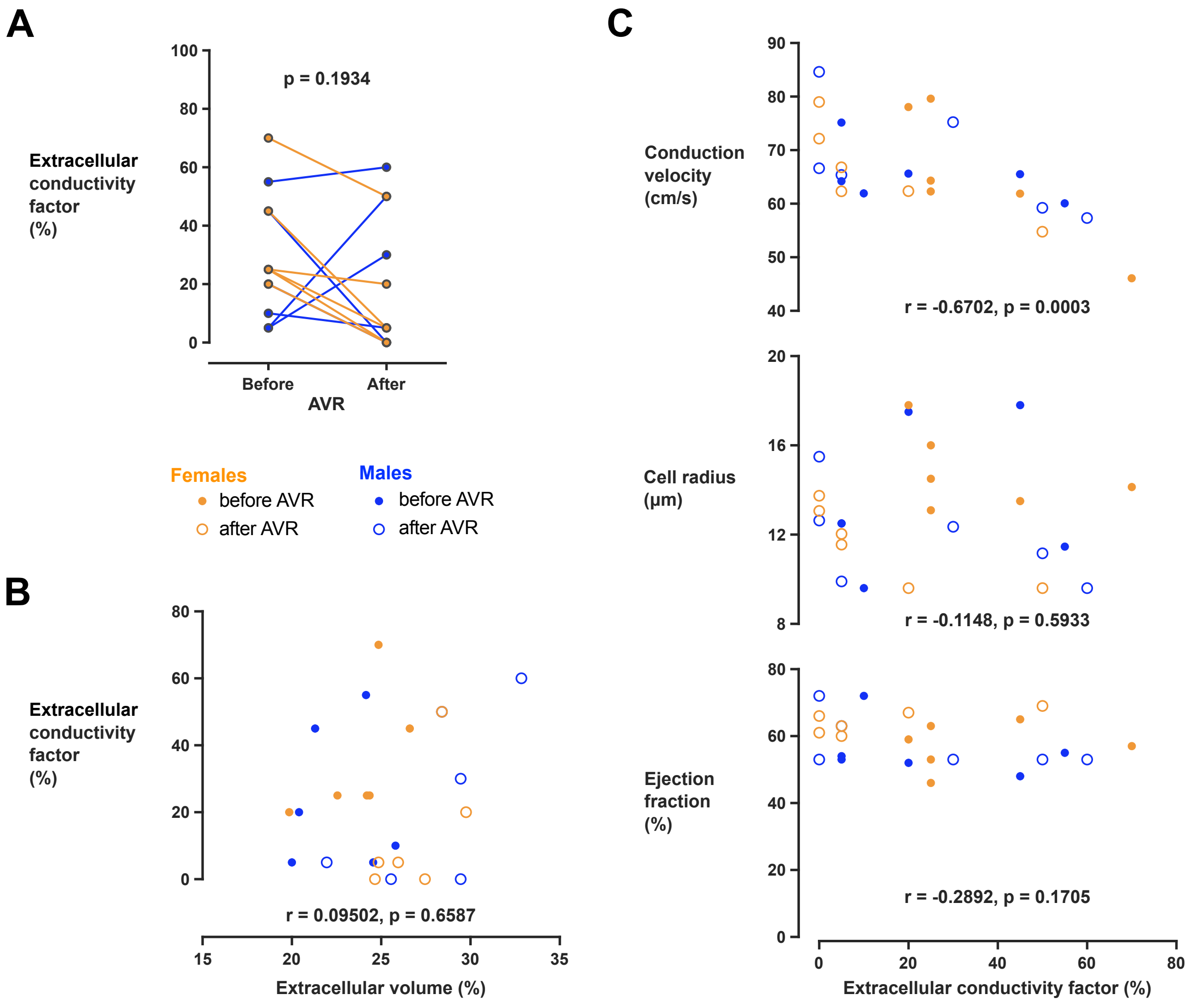

Figure S3: Extracellular conductivity factor (ECF). (A) No trend in the change of ECF between the pre-AVR and post-AVR time points was observed. **(B)** There was no association between extracellular volume (ECV) and ECF. **(C)** A negative correlation was found between ECF and conduction velocity. No correlation was found between ECF and cell radius, and ECF and ejection fraction.

2.5 Sensitivity analysis for the personalized tissue parameters

The effect of R, ECV and ECF on QRS duration and conduction velocity (CV) is illustrated in the sensitivity analysis presented in Figure S4. An increase in R, representing cellular hypertrophy, accelerates ventricular conduction and leads to shorter QRS duration. Similarly, an increase in ECV in absence of fibrosis leads to increased CV and shortening of QRS duration. In contrast, increased ECF leads to slower ventricular conduction and prolongs QRS duration.

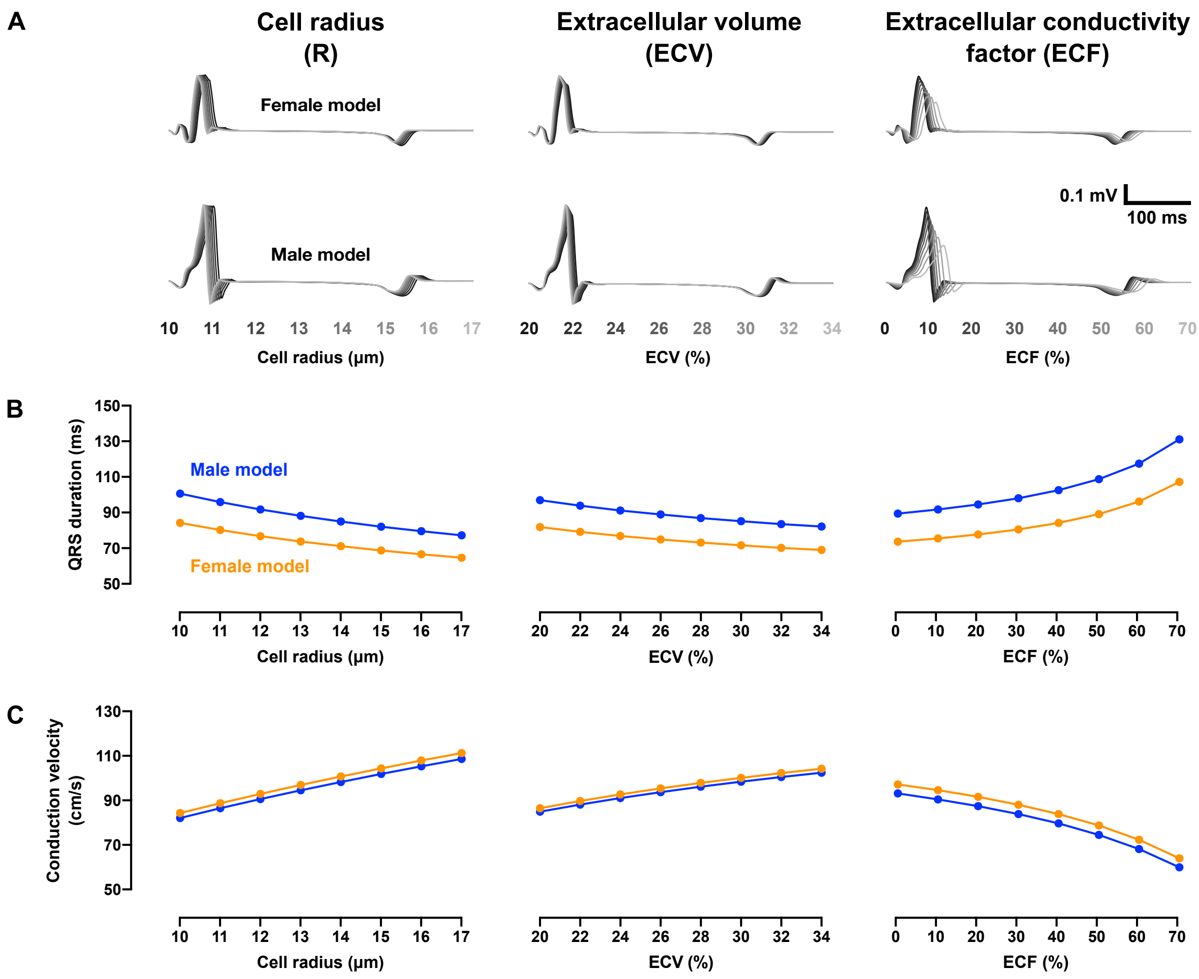

Figure S4: Sensitivity analysis illustrating the effect of cell radius (R), extracellular volume (ECV), and extracellular conductivity factor (ECF) on QRS duration and conduction velocity (CV). (A, B) An increase in R or ECV (in the absence of extracellular collagen content) shortens QRS duration while an increase in ECF results in longer QRS duration. **(C)** On the tissue level, R prolongation and an increase in ECV accelerate CV, while an increase in ECF decreases CV.

2.6 Sensitivity analysis for intracellular conductivity

The effect of intracellular conductivity (σ_i) on QRS duration and CV is shown in Figure S5. The sensitivity analysis shows that scaling σ_i in longitudinal direction ($\sigma_{i,l}$, direction of myocardial fibers) can considerably affect CV and QRS duration. In contrast, scaling σ_i in transversal ($\sigma_{i,t}$) and normal ($\sigma_{i,n}$) directions (the directions that are perpendicular to the direction of myocardial fibers) leads to modest changes in CV and QRS duration. The similarity between the data for $\sigma_{i,l}$ and simultaneous scaling of $\sigma_{i,l}$, $\sigma_{i,t}$, and $\sigma_{i,n}$ (overall change in σ_i) demonstrates that $\sigma_{i,l}$ plays a dominant role in intracellular conductivity, and that the effects of $\sigma_{i,t}$ and $\sigma_{i,n}$ on QRS duration are negligible. For comparison, Figure S5 also contains data on extracellular conductivity scaling presented in the sensitivity analysis in Figure S4. Only the data corresponding to ECF between 0 and 50 % have been used, so they correspond with the range of σ_i scaling applied in the sensitivity analysis.

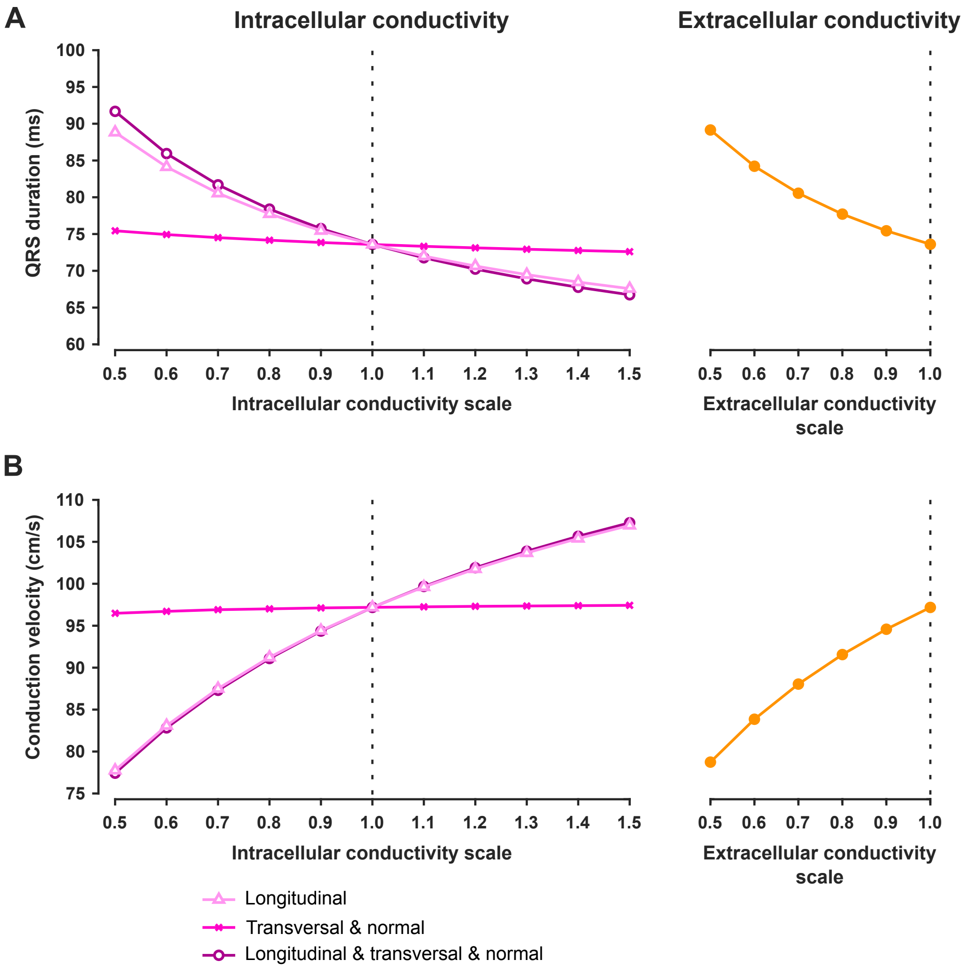

Figure S5: Sensitivity analysis for intracellular conductivity (σ_i). The effects of σ_i scaling in (a) longitudinal direction only ($\sigma_{i,l}$, the direction of myocardial fibers), (b) simultaneously in transversal and normal direction ($\sigma_{i,t}$, $\sigma_{i,n}$, respectively; the directions perpendicular to the myocardial fibers), and (c) simultaneously in longitudinal, transversal and normal direction (overall change in σ_i). **(A)** QRS duration markedly changed when σ_i was scaled, and when $\sigma_{i,l}$, $\sigma_{i,t}$, and $\sigma_{i,n}$ were simultaneously scaled. In contrast, the effect of simultaneous $\sigma_{i,t}$ and $\sigma_{i,n}$ scaling on QRS duration was very small. For comparison, the right panel shows the effect of extracellular conductivity on QRS duration. **(B)** Scaling $\sigma_{i,l}$ and simultaneous scaling of $\sigma_{i,l}$, $\sigma_{i,t}$, and $\sigma_{i,n}$ resulted in marked changes in conduction velocity. However, the effect of simultaneous $\sigma_{i,t}$ and $\sigma_{i,n}$ scaling was negligible. Again, the effect of extracellular conductivity scaling is provided for comparison. The values on the scale for extracellular conductivity correspond to ECF values ranging from 50 % (value 0.5) to 0 % (value 1).

3 Supplementary Discussion

3.1 The effect of ECV increase on CV and QRS duration

The sensitivity analysis presented in Figure S4 shows that an increase in ECV that is not accompanied by a reduction in extracellular conductivity due to the presence of fibrosis leads to an increase in CV and shortening of QRS duration. Such phenomenon can be seen as counter-intuitive and requires further explanation. For the bidomain equations, CV is proportional to

$$CV \propto \sqrt{\frac{\sigma_i \sigma_e}{\sigma_i + \sigma_e} \cdot \frac{1}{\beta}}, \tag{13}$$

where σ_i is intracellular conductivity, σ_e is extracellular conductivity, and β is the ratio of membrane area to tissue volume, obtained according to the equation 8. Under the conditions in which both σ_i and σ_e are constant, CV

becomes a function of cell radius (R) and ECV:

$$CV(R, ECV) \propto \sqrt{\frac{\sigma_i \sigma_e}{\sigma_i + \sigma_e} \cdot \frac{R}{2(1 - ECV)}}. \tag{14}$$

This illustrates that not only an increase in R, but also an increase in ECV can lead to increased CV.

CV is one of the key parameters that affects QRS duration: faster is the ventricular conduction, shorter is the QRS duration. Since increased ECV leads to faster CV, it also leads to shorter QRS duration, as illustrated in Figure S4.

3.2 Comparison of the results with previous studies

Figure S6 compares the cell diameter ($2R$) obtained in the personalized computational models with the results reported by Krayenbuehl et al. [8]. One can notice a similar degree of cell diameter shortening in both studies.

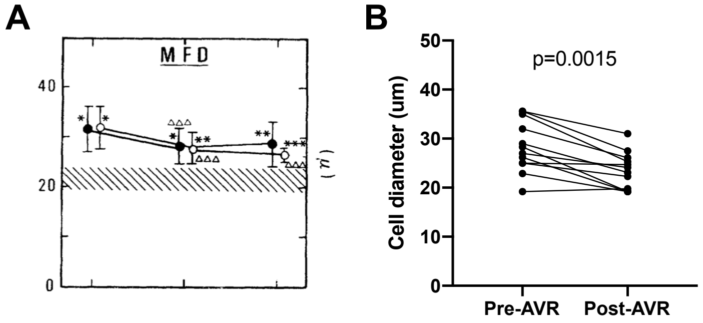

Figure S6: Comparison of the results reported by Krayenbuehl et al. and by this study. **(A)** Muscle fiber diameter (MFD) reported by Krayenbuehl et al. [8]. **(B)** Myocyte diameter acquired from the computational models reported in this study. Panel B corresponds with Figure 6C (cell radius) in the manuscript.

References

[1] Gillette K, Gsell MAF, Prassl AJ, Karabelas E, Reiter U, Reiter G, et al. A Framework for the generation of digital twins of cardiac electrophysiology from clinical 12-leads ECGs. Medical Image Analysis. 2021;71:102080. doi:10.1016/j.media.2021.102080.

[2] Merle M, Collot F, Castelneau J, Migerditichan P, Juhoor M, Ly B, et al. MUSIC: Cardiac Imaging, Modelling and Visualisation Software for Diagnosis and Therapy. Applied Sciences. 2022;12(12):6145. doi:10.3390/app12126145.

[3] Bayer J, Prassl AJ, Pashaei A, Gomez JF, Frontera A, Neic A, et al. Universal ventricular coordinates: A generic framework for describing position within the heart and transferring data. Medical Image Analysis. 2018;45:83–93. doi:10.1016/j.media.2018.01.005.

[4] Bayer JD, Lalani GG, Vigmond EJ, Narayan SM, Trayanova NA. Mechanisms linking electrical alternans and clinical ventricular arrhythmia in human heart failure. Heart Rhythm. 2016;13(9):1922–1931. doi:10.1016/j.hrthm.2016.05.017.

[5] ten Tusscher KHWJ, Noble D, Noble PJ, Panfilov AV. A model for human ventricular tissue. American Journal of Physiology-Heart and Circulatory Physiology. 2004;286(4):H1573–H1589. doi:10.1152/ajpheart.00794.2003.

[6] Cerqueira MD, Weissman NJ, Dilsizian V, Jacobs AK, Kaul S, Laskey WK, et al. Standardized myocardial segmentation and nomenclature for tomographic imaging of the heart. A statement for healthcare professionals from the Cardiac Imaging Committee of the Council on Clinical Cardiology of the American Heart Association. Circulation. 2002;105(4):539–542. doi:10.1161/hc0402.102975.

[7] Sobota V, Nordmeyer S, Augustin C, Plank G, Vigmond EJ, Bayer JD. A method for incorporating changes in extracellular volume and myocyte size into the cardiac bidomain equations. Computing in Cardiology. 2022;doi:10.22489/CinC.2022.203.

[8] Krayenbuehl HP, Hess OM, Monrad ES, Schneider J, Mall G, Turina M. Left ventricular myocardial structure in aortic valve disease before, intermediate, and late after aortic valve replacement. Circulation. 1989;79(4):744–755. doi:10.1161/01.CIR.79.4.744.

[9] Chin CWL, Everett RJ, Kwiecinski J, Vesey AT, Yeung E, Esson G, et al. Myocardial Fibrosis and Cardiac Decompensation in Aortic Stenosis. JACC: Cardiovascular Imaging. 2017;10(11):1320–1333. doi:10.1016/j.jcmg.2016.10.007.

[10] Poels TT, Houthuizen P, van Garsse LAFM, Hamad MAS, Maessen JG, Prinzen FW, et al. Frequency and prognosis of new bundle branch block induced by surgical aortic valve replacement. European Journal of Cardio-Thoracic Surgery. 2015;47:e47–e53. doi:10.1093/ejcts/ezu435.

[11] Durrer D, van Dam T, Freud GE, Janse MJ, Meijler FL, Arzbaecher RC. Total Excitation of the Isolated Human Heart. Circulation. 1970;41(6):899–912. doi:10.1161/01.cir.41.6.899.

[12] Sobota V, Nordmeyer S, Augustin C, Plank G, Vigmond EJ, Bayer JD. Overall fibrosis content rather than regional differences in extracellular volume alters ventricular conduction in aortic stenosis patients. Europace. 2023;25:euad122.263. doi:10.1093/europace/euad122.263.

[13] Treibel TA, Kozor R, Schofield R, Benedetti G, Fontana M, Bhuva AN, et al. Reverse Myocardial Remodeling Following Valve Replacement in Patients With Aortic Stenosis. Journal of the American College of Cardiology. 2018;71(8):860–871. doi:10.1016/j.jacc.2017.12.035.
